# Genetic Polymorphisms of Immunity Regulatory Genes and Alopecia Areata Susceptibility in Jordanian Patients

**DOI:** 10.3390/medicina60101611

**Published:** 2024-10-01

**Authors:** Mansour Alghamdi, Laith AL-Eitan, Hanan Aljamal, Hana Abu Kharmah

**Affiliations:** 1 Department of Anatomy, College of Medicine, King Khalid University, Abha 62529, Saudi Arabia; m.alghamdi@kku.edu.sa; 2 Genomics and Personalized Medicine Unit, The Centre for Medical and Health Research, College of Medicine, King Khalid University, Abha 62529, Saudi Arabia; 3 Department of Biotechnology and Genetic Engineering, Jordan University of Science and Technology, Irbid 22110, Jordan; haaljamal139@sci.just.edu.jo (H.A.); hsabokharmah20@sci.just.edu.jo (H.A.K.)

**Keywords:** alopecia areata, *BTNL2*, *CLEC4D*, *FASL*, *AIRE*

## Abstract

*Background and Objectives*: Alopecia areata (AA) is a tissue-specific immune-mediated disorder that affects hair follicles and the nail apparatus. Due to the collapse of hair follicle immune privilege in AA, hair loss ranges in severity from small, localized patches on the scalp to the loss of entire body hair. Although AA is of uncertain etiology, the disease has a common genetic basis with a number of other autoimmune diseases. *Materials and Methods*: To identify candidate genes that confer susceptibility to AA in the Jordanian population and further understand the disease background, we performed DNA genotyping using case–control samples of 152 patients and 150 healthy subjects. *Results*: While no significant result was observed in the ten single-nucleotide polymorphisms (SNPs), *CLEC4D* rs4304840 variants showed significant associations with AA development within our cohort (*p* = 0.02). The strongest associations were for the codominant and recessive forms of rs4304840 (*p* = 0.023 and *p* = 0.0061, respectively). *Conclusions*: These findings suggest that *CLEC4D* gene variants may contribute to AA pathogenesis among Jordanians. Further advanced genetic analysis and functional investigations are required to elucidate the genetic basis of the disease.

## 1. Introduction

Human alopecia areata (AA) is an autoimmune disease affecting specific organs, proposed to be caused by T-cell attacks of hair follicle (HF) immune privilege (IP) [1,2]. AA is a heterogeneous disease associated with a complex interaction between genetic, epigenetic, and environmental factors. A genetic component intertwined with other triggers is likely to determine disease susceptibility, onset, severity, duration, and recurrence [3,4]. The disease manifests as rapid-onset patchy hair loss in isolated or several well-defined round or oval patches. Alopecia totalis (AT) refers to complete scalp hair loss, while alopecia universalis is complete hair loss on the entire body [5,6]. AA is one of the most common immune hair loss disorders; it is equally distributed among all ethnic groups and genders and has a young age of onset, with a lifetime prevalence of 2% or higher [4,5,6,7,8].

Although most reported AA cases are sporadic, evidence from several studies suggests a complex polygenic nature of the disease [1]. Inherited predisposition components play a significant role in the disease’s pathology based on the increased positive family history among AA patients [2,3]. Genes that control both innate and adaptive immunity have been found to trigger AA development, and several AA susceptibility loci have also been obtained in other autoimmune diseases [4,5]. This study investigates several candidate genes associated with AA in a cohort of the Jordanian population, including the MHC class II gene-linked butyrophilin family member, butyrophilin-like 2 (*BTNL2*), autoimmune regulator (*AIRE*) gene, C-type lectin domain family 4 member D (*CLEC4D*), Complement Component 5 (C5), Fas cell surface death receptor (*FAS*), FAS ligand (*FASL*), killer cell immunoglobulin-like receptor, 3 Ig domains, and long cytoplasmic tail 1 (*KIR3DP*). The *AIRE* gene has a regulatory role on the autoreactive T cells in the thymus, whereas defects in the gene have been found to generate tissue-specific autoimmunity [6,7]. The Fas ⁄FasL system plays a crucial role in apoptosis and cellular homeostasis, besides its involvement in many human autoimmune and cancerous diseases [8,9]. Moreover, gene members of the complement system, *CTL*/*CTLD* superfamily, *KIR*, and *BTNL* gene cluster are responsible for controlling inflammation and immune response [10,11,12,13]. Primary association evidence has revealed multiple loci that influence AA susceptibility and severity near or within these immune-related genes, besides other types of autoimmunity [14]. 

## 2. Materials and Methods

### 2.1. Ethical Approval and Participants

This study was conducted under the guidelines of the Institutional Review Board (IRP) of Jordan University of Science and Technology (JUST), King Abdullah University Hospital (KAUH), Royal Jordanian Medical Services, and the Declaration of Helsinki. A total of 152 patients with AA were recruited from dermatology clinics in Jordanian Royal Medical Services (JRMS), Amman, Jordan. Patients were evaluated based on assessment guidelines for AA by Olsen et al., 2004 [15]. The age range was 13–67 years, with a mean age ± SD of 31.144 ± 12.41 for the 107 male and 45 female AA patients. One hundred fifty healthy controls with no clinical history of AA were enrolled from KAUH, Irbid, Jordan. The 129 male and 21 female controls had an age range of 17–64 years with a mean age ± SD of 33.9 ± 9.81. The basic criteria for inclusion were that the participants generally have good health and no first-degree relatives with autoimmune disorders. In addition, participants were required not to have any other autoimmune conditions. On the other hand, this study excluded individuals with a history of scalp or skin diseases that could confound the results (e.g., psoriasis, seborrheic dermatitis) or other acute or chronic debilitating diseases.

According to the National Alopecia Areata Foundation, 160 million people have alopecia areata, with a prevalence of 2% out of the worldwide population of 8 billion (https://www.naaf.org/alopecia-areata, accessed on 1 September 2024). OpenEpi software (Version 3.01., released on 4 April 2013) was used to calculate the required sample size with a 95% confidence interval. An alopecia areata prevalence of 2%, a design effect of 1, and a precision of 3% were considered while calculating the sample size, which was calculated as 84 participants. The study sample size exceeded the required 84 participants, with 152 individuals included in the case group.

### 2.2. SNP Selection and DNA Genotyping

Eleven single-nucleotide polymorphisms (SNPs) of interest within seven candidate genes that had been reported to have a possible or confirmed genetic association with AA were selected to be investigated in this study. These included *BTNL2* (rs28362679), *CLEC4D* (rs4304840), *FAS* (rs1800682 and rs2234767), *FASLG* (rs763110), *KIR3DP* (rs34531670 and rs597068), *AIRE* (rs1800520 and rs56393821), and *C5* (rs17611 and rs2230212). These SNPs were selected based on their biological significance, substantial functional effect, and location within the gene of interest. The functional annotations of the variants investigated in this study were further analyzed using HaploReg v4.2 (https://pubs.broadinstitute.org/mammals/haploreg/haploreg.php accessed on 1 September 2024). Functional annotation using HaploReg v4.2 indicated that *BTNL2* (rs28362679), *CLEC4D* (rs4304840), *KIR3DP* (rs34531670, rs597068), *AIRE* (rs1800520, rs56393821), and *C5* (rs17611, rs2230212) are mis-sense variants which can significantly affect protein function. In contrast, FAS (rs1800682 and rs2234767) are intronic variants, and *FASLG* (rs763110) is an upstream transcript variant (File S1). Furthermore, HaploReg v4.2 showed that these SNPs may indirectly contribute to the pathogenesis of alopecia areata through their potential effect on promoter and enhancer histone marks and alteration of transcription factor binding patterns.

Genomic DNA (gDNA) samples were extracted from blood samples collected in ethylenediaminetetraacetic acid (EDTA) using the Wizard^®^ Genomic DNA Purification Kit (Promega Corporation, Madison, WI, USA). The Sequenom MassARRAY^®^ (iPLEX GOLD) system (Agena Bioscience, San Diego, CA, USA). was used for genotyping in collaboration with the Australian Genome Research Facility (AGRF). After this, samples were amplified by PCR using designed primers to guarantee genotyping accuracy [16].

### 2.3. Statistical Analysis

The data were analyzed using the Statistical Package for Social Sciences (SPSS) version 26.0 (SPSS, Inc., Chicago, IL, USA). Allele and genotype frequencies related to the risk of AA among Jordanians were compared between the participant groups. The frequency distribution of the targeted genes between patients and controls was evaluated using the chi-square (χ^2^) test, with *p* values less than 0.05 considered significant. The odds ratio (OR) was used to calculate the risks associated with a specific genotype, providing a 95% confidence interval (CI) for the analysis. Additionally, SNPStats, a web-based tool, investigated various genetic models, haplotype frequency estimates, and Hardy–Weinberg equilibrium (https://www.snpstats.net/start.htm, accessed on 1 September 2024). The association between haplotypes and disease status was also investigated using SNPStats. Statistical significance was determined at a *p* value of less than 0.05. Moreover, a Bonferroni correction was performed, with the significance threshold set at α/n, where α equals 0.05 and *n* represents the number of tests [17]. This correction ensures that the overall *p*-value remains at a significance level of 0.005 or less.

## 3. Results

A total of 152 patients with AA were clinically diagnosed at a mean age of 31.144 ± 12.41 years; 87 patients (57.3%) experienced the onset of the disease early in life, before the age of 30 years. The clinical presentation of AA was mainly patchy AA (90.13%), followed by the less common severe AA forms: universalis and totalis (6.57% and 3.28%, respectively). The scalp was the most commonly affected site among patients (60.5%), although some presented with AA in the hair-bearing areas of the skin on the face (23.02%), including eyebrows, eyelashes, and beard, in addition to other targeted body sites (2.63%), such as the hair in the underarms and genital areas. Nail dystrophy was one of the risks and poor prognosis factors reported among 11 (7.3%) patients in this study. Disease-associated abnormalities manifested before the appearance of any hairless patches in 31.6% of our cohort of patients, while the remaining cases were asymptomatic.

The exact test for the Hardy–Weinberg equilibrium (HWE) showed that the variant distribution in the patients’ genotype was in equilibrium, except for *CLEC4D* rs4304840. Of the 11 SNPs of interest investigated in this study, only *CLEC4D* rs4304840 showed significant association (Table 1).

Although none of the controls had the allele ‘A’ of *BTNL2* rs28362679, compared to three AA patients, this difference did not reach any significance. Moreover, the GG genotype of *CLEC4D* rs4304840 revealed a possible association with disease development, since its incidence was almost duplicated in AA patients (23% vs. 11% in controls, *p* = 0.023). Among the genetic model of rs4304840, the codominant and recessive forms presented greater odds of association, with OR = 1.07 (95% CI: 0.65–1.76, *p* = 0.023) and OR = 0.42 (95% CI: 0.22–0.79, *p =* 0.0061), respectively, as presented in Table 2.

To further examine any possible association, we performed a haplotype analysis, which failed to confer genetic susceptibility to AA in Jordanian patients (Table 3). This study found no significant association between any SNPs and the disease after applying the Bonferroni correction with a significance level of *p* = 0.005.

## 4. Discussion

The current investigation is a continuum study of AA susceptibility genes in the Jordanian population [16,18,19,20]. AA is among the most highly prevalent autoimmune hair loss disorders, affecting humans of all ages, genders, and races [21]. Current progress in genetic research has identified several candidate genes associated with AA development. Most of these genes significantly affect immune system regulation and autoimmunity [5,14]. Among these is the *BTNL2* gene, which inhibits T cell activation and signaling pathways [4,13]. The regulatory function of *BTNL2* on T cell proliferation may explain the possible association of this gene variant with inflammatory and autoimmune diseases, including AA [13,22]. According to genome-wide association studies (GWASs) and previous candidate gene studies, this gene is involved in Crohn’s disease (CD), systemic lupus erythematosus (SLE), generalized vitiligo (GV), multiple sclerosis (MS), rheumatoid arthritis (RA), Graves’ disease (GD), ulcerative colitis (UC), and type 1 diabetes (T1D). While GWASs in AA highlighted *BTNL2* as a general AA susceptibility gene [22], recent exome sequencing of immune-related genes revealed *BTNL2* rs28362679 among the most significantly associated SNPs (*p* < 0.001 and OR = 30.21) [23]. This polymorphism was not a statistically significant SNP in this study, in contrast to previous studies that suggested *BTNL2* conferred susceptibility to AA.

The highly significantly associated SNP in the present cohort is *CLEC4D* rs4304840. This result contradicts a previous finding: rs4304840 failed to be associated with AU in the case–control analysis conducted by Lee et al., 2013 (*p* = 0.06) [23]. The *CLEC4D* gene is critical in infectious diseases, such as pulmonary tuberculosis (TB), as mycobacterial recognition is one of its vital functions. Data suggested that mutations in this gene can reduce surface expression, increasing TB susceptibility [24,25]. Although this gene belongs to the C-type lectin receptor family, an integrated part of innate immunity, and has a crucial role in immune homeostasis [26], it has rarely been studied in association with AA.

AA is an autoimmune disease caused by T cells attacking hair follicles expressing FAS and its ligand (*FASLG*) on the perifollicular infiltrates [9,27]. The *FAS*/*FASLG* system induces the positive regulation of apoptosis and maintains cellular homeostasis. Therefore, abnormal interactions of these molecules and the resulting signaling pathways have a significant role in autoimmune diseases and cancer pathogenesis [28,29,30,31,32]. Polymorphisms including rs1800682, rs2234767, rs763110, and rs5030772 within *FAS*/*FASLG* genes have previously been examined for their potential association with AA in different subjects from Chinese, Turkish, Iranian, and Egyptian populations. In the latter cohorts, a significant association was reported for *FASLG* rs5030772 but not *FAS* rs1800682 [33], agreeing with the findings of Tabatabaei-Panah et al., 2019 [34]. In contrast, *FAS* rs1800682 was significantly distributed among Turkish participants, and observations suggested that this variant could be protective against AA, whereas *FASLG* rs5030772 has no risk associated with the disease [8]. Similarly, *FAS* rs1800682 has a reduced association with disease susceptibility, compared to the lack of association found for FAS rs2234767 and *FASLG* rs763110 [9]. Regarding the findings among the Jordanian population, data analysis also revealed no association between *FAS* rs2234767 and rs1800682 and *FASLG* rs763110 with AA genetic predisposition.

The autoimmune regulator (*AIRE*) is another candidate immune-related gene that develops multiple tissue-specific autoimmunities by inducing adverse selection of T cells in the thymus [2,6]. Mutations in the *AIRE* gene result in autoimmune polyendocrine syndrome type 1 (APS1), which presents with several clinical manifestations, including alopecia, one of the most frequently associated autoimmune disorders with the syndrome [7,35,36,37,38,39]. These findings provide evidence of the potential involvement of *AIRE* in the pathogenesis of AA among the studied *AIRE* SNPs, rs56393821, that did not support the hypothesis that *AIRE* mutations are strongly predisposed to alopecia in samples of British [37], Belgian-German [40], and Jordanian origin [current study]. This is the case for the rs1800520 with AA [38,40], while based on other screening data, it was found to be associated with the severest form (AU) of AA and early disease onset [37]. *AIRE* rs56393821 and *C5* rs2230212 (previously rs75268750) SNPs are monomorphic in Jordanians, which seems to be unique, as to the best of our knowledge, these variants are polymorphic in other populations. For instance, *C5* rs2230212 and rs17611 were shown to be genetic risk components for developing AU in a whole-exome sequencing analysis [23]. Contrastingly, these two SNPs did not influence susceptibility to AA and were not associated with positive family history in a large case–control study of Central European patients (1195 patients and 1280 controls) [41,42], which is consistent with our findings. The killer-cell immunoglobulin-like receptor (*KIR*) genes control the activation and function of natural killer (NK) cells by interacting with human leukocyte antigen (HLA) class I ligands [43].

As NK has an essential role in the defense mechanisms of the female reproductive system, any genetic variation in the *KIR* receptors expressed on the cell surface may result in health concerns. Polymorphisms in KIRs and their HLA ligands were shown to be associated with the development of polycystic ovary syndrome (PCOS) in Brazilian females [42]. Thus, *KIRs* are likely to have a vital role in immune response modulation, which would explain their associations with rheumatoid arthritis (RA) [44,45] and psoriatic arthritis (PsA) [46]. As a result, some candidate loci within the KIR3DP gene have been analyzed for possible association with AA risk; rs34531670 and rs597068 (merged from rs77919587 and s116897146, respectively) lack any observed risk with the disease [23] and the current study]. The data indicate that the onset of alopecia areata (AA) predominantly occurs before the age of 30 in the majority of patients (57.3%), suggesting that AA frequently manifests in younger individuals. However, the absence of statistically significant differences in age and gender implies no evidence from this dataset to support the notion that younger patients are more likely to exhibit distinct genetic alterations associated explicitly with age. Although the early onset of AA may suggest a potential role for genetic factors being more prominent in younger individuals, the current analysis does not substantiate this hypothesis.

Further studies, particularly those incorporating genomic analyses or family history assessments, are required to elucidate potential age-related genetic variations. Moreover, other genes that are possibly involved in in AA development or treatment should be investigated; for example, the tumor suppressor p53, a frequently mutated gene in human cancer, is a promising target for anticancer immunotherapies [47]. Future studies should investigate the relationship between p53 and alopecia areata, highlighting the potential link between immune dysregulation in both diseases.

## 5. Conclusions

Our results point to the contribution of the *CLEC4D* gene to the onset of AA in the Jordanian population and elucidate an additional emphasis on the genetic basis of the disease, the role of immune-related genes in its etiology, and the risk SNPs shared with other autoimmune diseases. Nevertheless, our study fails to confirm the association of *FAS*, *FASLG*, *AIRE*, *C5*, and *KIR3DP* with susceptibility to AA. Functional analysis, in addition to clinical genetic correlation, is required for our future understanding of the molecular pathogenesis of AA.

## Figures and Tables

**Table 1:** Allele and genotype frequency distribution of gene polymorphisms in AA patients and healthy controls.

Gene	SNP	Allele/Genotype	Control (*n* = 150) (*n*, %) ^a^	AA Patients ^b^ (*n* = 152) (*n*, %) ^a^	*p*-Value ^c^
*BTNL2*	rs28362679	G	296, 100	301, 99	0.08
		A	0, 0	3, 1	
		GG	148, 100	149, 98	0.08
		GA	0, 0	3, 2	
*CLEC4D*	rs4304840	A	191, 65	171, 57	0.04
		G	103, 35	129, 43	
		AA	60, 41	55, 37	0.02
		AG	71, 48	61, 41	
		GG	16, 11	34, 23	
*KIR3DP*	rs34531670	T	254, 86	255, 85	0.77
		C	42, 14	45, 15	
		CC	4, 3	2, 1	0.5
		TC	34, 23	41, 27	
		TT	110, 74	107, 71	
	rs597068	G	251, 85	254, 84	0.81
		C	45, 15	48, 16	
		CC	4, 3	1, 1	0.24
		CG	37, 25	46, 3	
		GG	107, 72	104, 69	
*AIRE*	rs56393821	T	296, 100	304, 100	Monomorphic
	rs1800520	C	260, 89	267, 88	0.64
		G	32, 11	37, 12	
		CC	115, 79	117, 77	0.82
		CG	30, 21	33, 22	
		GG	1, 1	2, 1	
*C5*	rs2230212	C	296, 100	304, 100	Monomorphic
	rs17611	C	175, 60	169, 56	0.37
		T	119, 40	133, 44	
		CC	51, 35	51, 34	0.36
		CT	73, 50	67, 44	
		TT	23, 16	33, 22	
*FAS*	rs1800682	A	162, 55	162, 54	0.72
		G	132, 45	140, 46	
		AA	46, 31	45, 30	0.93
		AG	70, 48	72, 48	
		GG	31, 21	34, 23	
	rs2234767	G	264, 89	268, 88	0.69
		A	32, 11	36, 12	
		AA	4, 3	2, 1	0.41
		GA	24, 16	32, 21	
		GG	120, 81	118, 78	
*FASLG*	rs763110	T	151, 51	169, 56	0.29
		C	143, 49	135, 44	
		CC	37, 25	26, 17	0.2
		TC	69, 47	83, 55	
		TT	41, 28	43, 28	

^a^ *n*, number and %, frequency; ^b^ AA, alopecia areata; ^c^ Significant *p*-values are shown in bold face. *p*-values < 0.005 (0.05/# of SNPs, 0.05/10 = 0.005 after applying multiple comparisons) are considered significant.

**Table 2:** Selection of the best genetic model for gene variant *CLEC4D* rs4304840.

Model	Genotype	Controls (*n*, %) ^a^	AA Patients (*n*, %)	OR (95% CI) ^b^	*p*-Value ^c^
Codominant	A/A	60 (40.8%)	55 (36.7%)	1.00	0.023
	G/A	71 (48.3%)	61 (40.7%)	1.07 (0.65–1.76)	
	G/G	16 (10.9%)	34 (22.7%)	0.43 (0.21–0.87)	
Dominant	A/A	60 (40.8%)	55 (36.7%)	1.00	0.46
	G/A-G/G	87 (59.2%)	95 (63.3%)	0.84 (0.53–1.34)	
Recessive	A/A-G/A	131 (89.1%)	116 (77.3%)	1.00	0.0061
	G/G	16 (10.9%)	34 (22.7%)	0.42 (0.22–0.79)	

^a^ *n*, number and %, frequency; ^b^ OR, odds ratio; CI, confidence interval; ^c^ significant *p*-values are shown in bold face. *p*-values < 0.005 (0.05/# of SNPs, 0.05/10 = 0.005 after applying multiple comparison) are considered significant.

**Table 3:** Haplotype analysis of *FAS* and *KIR3DP* gene polymorphisms in the study cohort.

Gene	Haplotype	Total	Controls	AA Patients	OR (95% CI)	*p*-Value
*FAS*	AG	0.5431	0.5494	0.5369	1.00	---
	GG	0.3436	0.3425	0.3447	0.97 (0.69–1.37)	0.87
	GA	0.1133	0.1081	0.1184	0.90 (0.54–1.49)	0.68
*KIR3DP*	TG	0.8377	0.8445	0.8309	1	---
	CC	0.1402	0.1385	0.1418	0.96 (0.60–1.55)	0.87
	TC	0.0153	0.0136	0.0171	0.81 (0.24–2.74)	0.74

Global haplotype association *p*-values for *FAS* and *KIR3DP* are 0.92 and 0.84, respectively. *p*-values < 0.005 (0.05/# of SNPs, 0.05/10 = 0.005 after applying multiple comparison) are considered significant.

## Data Availability

The raw data supporting the conclusions of this article will be made available by the authors on request.

## Supplementary Materials

The following supporting information can be downloaded at: https://www.mdpi.com/article/10.3390/medicina60101611/s1, File S1: Genotypic data for patients and control, Cluster Plots, and Primer Sequences.
